# Could Lowering Phytosterol Absorption as Part of Lipid-Lowering Therapy Have a Beneficial Effect on Residual Risk?

**DOI:** 10.3390/metabo13020145

**Published:** 2023-01-18

**Authors:** Panagiotis Anagnostis, Vasileios Kotsis, Maciej Banach, Dimitri P. Mikhailidis

**Affiliations:** 1 3rd Department of Internal Medicine, Medical School, Aristotle University of Thessaloniki, “Papageorgiou” General Hospital Thessaloniki, 56429 Thessaloniki, Greece; 2 Department of Preventive Cardiology and Lipidology, Medical University of Lodz (MUL), 90-419 Lodz, Poland; 3 Department of Cardiology and Adult Congenital Heart Diseases, Polish Mother’s Memorial Hospital Research Institute (PMMHRI), 93-338 Lodz, Poland; 4 Cardiovascular Research Centre, University of Zielona Gora, 65-417 Zielona Gora, Poland; 5 Department of Clinical Biochemistry, Royal Free Hospital Campus, University College London Medical School, University College London (UCL), London NW3 2QG, UK

**Keywords:** phytosterols, sitosterol, campesterol, statins, ezetimibe

## Abstract

Plant sterols are molecules that are structurally similar to cholesterol and provided only as dietary sources (e.g., vegetables, fruits, nuts, cereals) since they cannot be synthesized by humans. Sterol-enriched diets (≥2 g/day) may decrease total and low-density lipoprotein cholesterol concentrations by 5–10%, either alone or when added to statins, since they antagonize dietary cholesterol absorption in the intestine. On the other hand, increased serum phytosterol concentrations, (including when associated with sitosterolemia, a rare genetic defect) may contribute to atherosclerotic risk, although a threshold for such a role has not been established. Medications such as ezetimibe may effectively reduce cholesterol and phytosterol absorption. Whether the therapeutic approach associated with the reduction of phytosterol absorption is also translated into a reduction in a patient’s residual cardiovascular risk needs to be established.

## 1. The Role of Phytosterols in Cholesterol Metabolism

Cholesterol homeostasis is tightly regulated by the interplay between endogenous synthesis, exogenous absorption and excretion (bile acids) [1]. Cholesterol absorption is regulated by the sterol influx membrane transporter Niemann-Pick C1-Like 1 (NPC1L1) and annexin 2/caveolin 1(ANXA2-CAV1) complexes, in enterocytes and hepatocytes [2]. NPC1L1 in the liver is also essential for the uptake of oxysterols, the oxidized products of cholesterol, which are enzymatically produced during cholesterol catabolism (e.g., 27-hydroxycholesterol) or non-enzymatically generated (e.g., 7-ketocholesterol). NPC1L1 also suppresses oxysterol biliary excretion [3]. Moreover, in vitro and in vivo studies suggest that 27-hydroxycholesterol and 25-hydroxycholesterol, which are NPC1L1 substrates, are also involved in hepatic steatosis progression, through activation of the liver X receptor (LXR)-alpha and retinoid-related orphan receptor gamma, respectively [3]. 

**Figure 1:**
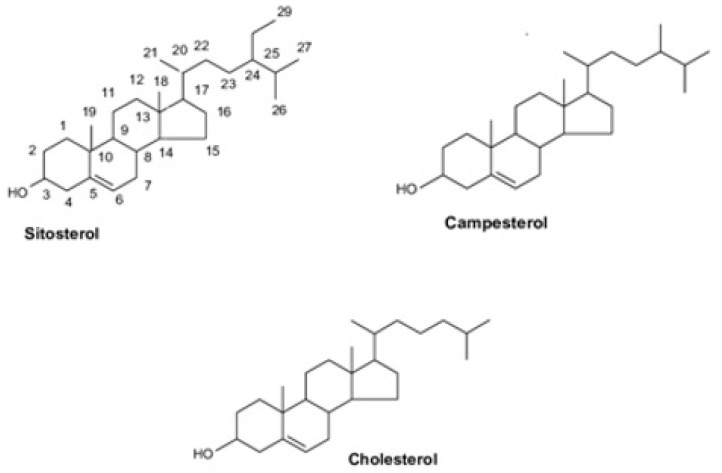
Chemical structure of cholesterol, sitosterol and campesterol.

Cholesterol metabolism is also determined by its secretion by ATP-binding cassette (ABC) transporters located on the brush border of intestinal cells [1,2]. Serum plant sterols or phytosterols have been used as markers of cholesterol absorption [2]. These sterols, as well as their saturated forms, termed phytostanols, are structurally similar to cholesterol (Figure 1) but cannot be synthesized by humans. They are only provided from dietary sources, mainly plant foods (vegetables, fruits, nuts, cereals). Sitosterol, campesterol and stigmasterol account for the vast majority of the total phytosterol dietary intake [2].

According to a large American cohort (*n* = 667,718), mean plasma concentrations for sitosterol and campesterol are 2.45 ± 1.39 and 3.3 ± 1.83 μg/mL, respectively [4]. This is similar in other populations. For example, the respective concentrations in Japanese males and females are 0.99–3.88 and 1.03–4.45 μg/mL [5]. Phytosterol levels are largely affected by age, gender and apolipoprotein E (apoE) genotype [4]. In particular, sitosterol concentrations are higher in women than in men during the 4th and 5th decade of life, but there is a marked and progressive increase thereafter, significantly exceeding the male pattern, which remains stable [4]. This is also the case with campesterol, the levels of which show an abrupt decrease after the 6th decade in males and a blunter decrease in females. Moreover, carriers of the *APOE* ε2 allele tend to have lower levels of these sterols compared with the *APOE* ε3 homozygotes, whereas carriers of the *APOE* ε4 allele tend to have higher concentrations [4].

Consumption of plant sterols has been recommended as a part of a well-balanced diet in order to reduce serum cholesterol concentrations, especially in patients at high risk of cardiovascular disease (CVD) or those with statin intolerance [6,7,8,9]. Plant sterols may decrease intestinal cholesterol absorption by competing with dietary and biliary cholesterol for its incorporation into mixed micelles [6]. Except for their interference in the absorptive process, a direct hypocholesterolemic effect of phytosterols/phytostanols inside the enterocyte or hepatocyte has been demonstrated. In particular, they can act either at LXR-mediated targets, such as the ABC transporter A1 (*ABCA1*), and subfamily G member 5 (*ABCG5*) and 8 (*ABCG8*) genes, or at LXR-independent targets such as apolipoprotein B (apoB), ANXA2 and the enzymes hydroxymethylglutaryl-CoA (HMG-CoA) reductase and acyl-CoA:cholesterol acyltransferase (ACAT) [2,10]. ACAT 2 is responsible for the esterification of intracellular cholesterol in the endoplasmic reticulum, which is packed into chylomicrons and excreted into the lymph system through the basolateral membrane [2,10]. LXR-dependent targets are shared by both stanols and sterols, whereas apoB, ANXA2, HMG-CoA reductase and ACAT may be affected only by phytosterols [2].

Unlike cholesterol, sterols cannot be esterified, although they enter into the enterocyte (via the NPC1L1 transporter) and are pumped back into the intestinal lumen via the *ABCG5/ABCG8* heterodimer [10]. Therefore, only 0.4–3.5% of dietary plant sterols are absorbed under normal conditions, while the remainder are returned to the intestinal lumen, in contrast to cholesterol (35–70% of the ingested amount is absorbed). Phytostanol absorption is even lower (0.02–0.3%) [2]. This small amount of absorbed plant sterols and stanols is excreted into the hepatobiliary system via the *ABCG5/ABCG8* heterodimer, resulting in relatively low levels in the circulation (1000-fold lower than cholesterol) [10].

However, ingestion of plant sterols may decrease low density lipoprotein (LDL) cholesterol (LDL-C) concentrations by 12.1 mg/dL (0.32 mmol/L), an effect which is more evident with daily doses ≥ 2 g/day and in patients with LDL-C ≥ 140 mg/dL (3.68 mmol/L) [11]. The same reductions in total cholesterol and LDL-C are achieved when sterol-enriched diets are added to statins, although they have no effect on triglyceride (TG) and high-density lipoprotein cholesterol (HDL-C) levels [12]. However, the exact association of their serum concentrations with an individual’s CVD risk has not been fully elucidated.

## 2. Phytosterols and Cardiovascular Risk

Sitosterolemia, a rare autosomal recessive disorder of lipid metabolism (OΜΙΜ #210250 and #618666), is characterized by markedly elevated plant sterol concentrations in blood (e.g., sitosterol, campesterol and sigmasterol) due to increased intestinal absorption and decreased biliary secretion [13,14]. This is due to homozygous or compound heterozygous loss-of-function mutations of the *ABCG5* and *ABCG8* genes, which limit sterol absorption from the intestinal epithelium into the lumen [13,14]. The prevalence of homozygous/compound heterozygous sitosterolemia in the general population is 1 in 200,000. Interestingly, some patients with familial hypercholesterolemia (FH) may be misdiagnosed, since more than half of those with the monogenic form may carry mutations of the *ABCG5* or *ABCG8* gene, mimicking or exacerbating their clinical phenotype [15]. Serum sitosterol concentrations are usually >1 mg/dL (10 μg/mL). In general, plasma total cholesterol and LDL-C levels are usually normal or modestly elevated [14]. Severe hypercholesterolemia has also been reported [16].

Patients with sitosterolemia present with cutaneous or tendon xanthomas and mostly with premature atherosclerotic CVD (males < 45 years; females < 50 years), resembling FH [14]. However, the role of sitosterol in the development of atherosclerosis has not been clarified. It is also unknown if sitosterol per se or increased LDL-C levels are responsible for premature CVD in these patients [14]. They may also manifest with macrothrombocytopenia leading to severe bleeding episodes as well as splenomegaly and hemolytic anemia, due to the abnormal accumulation of sitosterol in blood cell membranes which compromises their morphology and function [14,17].

Apart from the rare cases of sitosterolemia, the role of phytosterol concentrations on CVD risk in the general population has also been assessed. Higher sitosterol concentrations [18] and a higher campesterol-to-cholesterol ratio [19] have been reported in patients with coronary heart disease (CHD) compared with those without CHD [20]. However, others did not find any difference in cholesterol absorption markers between these groups [21]. Furthermore, a meta-analysis of 17 studies (four case–control, three cohort, five cross-sectional and five nested case–control studies), including 11,182 participants in total, did not show any association between sitosterol and campesterol concentrations and the risk of atherosclerotic CVD. However, there was evidence for publication bias and substantial heterogeneity among studies [22]. More recent data indicate a positive association of increased cholesterol absorption, as expressed by the campesterol-to-cholesterol ratio, with increased risk of in-stent restenosis in patients with stable CHD [23]. Another recent cross-sectional study (*n* = 270 asymptomatic individuals) showed a positive association of carotid intima-media thickness with serum campesterol concentrations and an inverse association between both lathosterol/campesterol and lathosterol/sitosterol ratios [24]. These findings suggest an association between carotid atherosclerotic plaques and campesterol levels, and an inverse association with synthesis/absorption ratios [24].

On a genetic basis, the *ABCG5/ABCG8* genes involved in sterol excretion into the intestinal lumen are associated with a higher CVD risk compared with those involved in cholesterol synthesis, such as the LDL-receptor (*LDLR*), *APOB* and HMG-reductase genes. Briefly, a genetic score of *ABCG5/ABCG8*, variants which predict a 1 mmol/L (39 mg/dL) increase in non-HDL-C, is associated with 2-fold increase in risk of coronary heart disease (CHD) [odds ratio (OR) 2.01, 95% confidence interval (CI) 1.75–2.31]. This is higher than that predicted by other genetic variants outside the *ABCG5/ABCG8* locus, causing the same increase in non-HDL-C (OR 1.54, 95% CI 1.49–1.59) [25]. This clearly supports an atherogenic role of the *ABCG5/ABCG8* variants that is not mediated through non-HDL-C.

All these data question the “beneficial” effect of pharmacological supplementation with plant sterols and stanols which impair the intestinal absorption/re-absorption of cholesterol, in terms of CVD risk. Furthermore, no randomized-controlled trials (RCTs), have been conducted so far, which assess a potential effect of phytosterol supplementation on hard clinical CVD endpoints of atherosclerosis [26].

## 3. The Effect of Hypolipidemic Treatment on Phytosterol Concentrations

With regard to sitosterolemia, dietary cholesterol and plant sterol restriction constitute the mainstay of treatment, aiming at an LDL-C target as in patients with FH (<70 mg/dL; 1.8 mmol/L). Ezetimibe, which acts by blocking NPC1L1, reduces the intestinal absorption of cholesterol. This, in turn, increases the expression of the LDL receptor in hepatocytes, resulting in reductions in LDL-C levels by about 20% [27,28].

In addition, ezetimibe can reduce serum sitosterol levels by 20–35%, as well as red blood cell sitosterol accumulation (−28%) [14,29]. It may also increase the platelet count (+23%) and decrease mean platelet volume (−10%) [29]. Ezetimibe may also be beneficial in patients under parenteral nutrition with plant-based lipid emulsion containing high levels of phytosterols, although relative studies are lacking. In these cases, addition of omega-3 fatty acids may protect against the adverse effects of phytosterols, as shown recently [30]. Bile-acid sequestrant resins may also reduce serum sitosterol by 30%. LDL apheresis is another option in patients with sitosterolemia who are unresponsive to other interventions [14]. However, so far there is no evidence that lowering sitosterol levels results in a reduction in CVD risk.

On the other hand, statins can only be considered in the setting of secondary prevention, since they can raise phytosterol levels. Statins downregulate cholesterol production in the liver, decreasing its blood levels by 30–50%, depending on dose and potency [31]. However, this leads to a compensatory increase intestinal cholesterol absorption [31]. Indeed, statins increase cholesterol absorption markers, such as campesterol and sitosterol [21,32], an effect which varies across different types of statin, ranging from 50% and 25%, respectively, with pravastatin 40 mg/day, 52% and 67% with rosuvastatin 40 mg/day, and 72% and 96%, with atorvastatin 80 mg/day [32]. Simvastatin 20–40 mg/day may also increase cholesterol absorption by 49% with a 30% increment in campesterol and sitosterol concentrations, respectively; this effect is negatively associated with baseline plasma glucose levels [33].

On the other hand, ezetimibe decreases phytosterol absorption, as mentioned above. Ezetimibe can also reduce triglyceride (TG) and non-HDL-C concentrations, although to a lesser extent when compared with statins [27,28]. Ezetimibe may either increase or decrease HDL-C levels [27,28]. The effect of ezetimibe on LDL-C, non-HDL-C and cholesterol absorption markers may be more prominent in the presence of type 2 diabetes mellitus (DM) [34]. Moreover, ezetimibe favorably alters the distribution of LDL by decreasing the concentrations of small, dense LDL particles. This effect is more pronounced in patients with high TG (i.e., 1.7 mmol/L or 150.4 mg/dL) compared with those with lower levels (49 vs. 18%, *p* < 0.05) [28]. International guidelines on the management of dyslipidemias recommend the addition of ezetimibe if the LDL-C target is not achieved with a maximally tolerated statin dose, or in patients with statin intolerance [6]. The combination of ezetimibe with plant sterols may decrease LDL-C levels by 25% compared with placebo, although this decrease is not different from that observed with ezetimibe alone (−22%) [35].

The combination of statin with ezetimibe is more efficacious compared with a statin alone [36,37], as it offers a dual effect of inhibiting cholesterol synthesis (with statin, which also decreases systemic inflammation) and cholesterol and sitosterol absorption (with ezetimibe) [38,39]. Post hoc analysis of an RCT including 874 patients with hypercholesterolemia showed that those on a high-potency statin demonstrated lower synthesis (as indicated by lathosterol levels) and higher absorption (as indicated by sitosterol levels) compared with those on a moderate- or low-potency statin [40]. Interestingly, the addition of ezetimibe provided a greater reduction in LDL-C concentrations in the former than in the latter groups (−29.1 vs. −25 vs. 22.7%, respectively) [40].

The combination of statins with ezetimibe further reduces CVD risk in patients with established CVD compared with statin monotherapy, as shown in the Improved Reduction of Outcomes: Vytorin Efficacy International Trial (IMPROVE-IT) [41]. Briefly, 18,144 patients with acute coronary syndrome (ACS) and moderately elevated LDL-C concentrations (of up 100–120 mg/dL; 2.6–3.2 mmol/L) were randomized to either simvastatin monotherapy (40 mg/day) or to combination with ezetimibe (10 mg/day). After 7 years of follow-up, the combination therapy decreased LDL-C [53 mg/dL (1.4 mmol/L) compared with 70 mg/dL (1.8 mmol/L) with statin monotherapy], which was translated into lower rates of CVD events [hazard ratio (HR) 0.93, 95% CI 0.88–0.99] [41]. Post-hoc analysis of another RCT showed that ezetimibe added to statin reduces the risk of in-stent restenosis in patients with stable CHD, compared with statin monotherapy [23]. A very recent study using intravascular ultrasound (IVUS) evaluation showed greater regression in plaque formation with the combination of a statin and ezetimibe than statin alone (any statin was used and titrated to achieve an LDL-C level of <100 mg/dL). This was mainly due to decreased cholesterol absorption, as indicated by the reduction in campesterol concentrations, as well as that of 27-hydroxycholesterol, rather than to a reduction in LDL-C levels [42].

Inconsistent with the IMPROVE-IT study, the Heart Institute of Japan-PRoper level of lipid lOwering with Pitavastatin and Ezetimibe in acute coRonary syndrome (HIJ-PROPER) study, did not show any CVD benefit with the combination of pitavastatin plus ezetimibe therapy, compared with pitavastatin alone, in patients with ACS [41]. However, a post hoc analysis of the HIJ-PROPER trial showed that, despite higher LDL-C concentrations in the high sitosterol group (≥2.2 μg/mL), there was a lower rate of CVD events in the combination therapy group compared with pitavastatin monotherapy group (HR 0.71, 95% CI 0.56–0.91). This difference was not observed in the low sitosterol group (<2 μg/mL), suggesting that sitosterol assessment may contribute to a more individualized lipid-lowering approach [43]. This was also confirmed in the Scandinavian simvastatin survival study (4S), in which an increased cholestanol:cholesterol ratio (an index of high absorption of cholesterol) compromised the beneficial effect of simvastatin on CVD risk (the higher the ratio, the greater the risk of major coronary events, regardless of the reduction in cholesterol concentrations) [44]. In accordance with the HIJ-PROPER and the 4S, another RCT in patients with atherosclerotic CVD failed to show the superiority of the combination of rosuvastatin 10 mg/day plus ezetimibe 10 mg/day compared with rosuvastatin 20 mg/day in terms of CVD risk reduction (absolute difference in 3-year CVD risk −0.78%; 90% CI −2.39% to 0.83%). However, a greater proportion of patients achieved LDL-C levels <70 mg/dL (1.8 mmol/L) with the combination therapy at 1, 2 and 3 years, compared with rosuvastatin monotherapy (73%, 75% and 72% vs. 55%, 60% and 58%, respectively; all *p* < 0.001). Moreover, a greater proportion of patients discontinued statin monotherapy due to intolerance compared with those on the combination regimen (8.2% vs. 4.8%, respectively; *p* < 0.0001) [45].

The beneficial effect of the combination of statin plus ezetimibe on CVD risk compared with statin alone, warrants further investigation. Apart from an additive (or even synergistic effect) on lipid profile, the decrease in phytosterol absorption with ezetimibe may also turn out to be a contributor to a better clinical outcome. Furthermore, experimental data suggest a favourable effect on glucose homeostasis, as well as increased fatty acid oxidation and reduced adipocytic inflammation [46]. At a clinical level, in contrast to the diabetogenic effect of statins, ezetimibe has a rather neutral or beneficial effect on glucose metabolism either in patients with or without DM [47,48].

## 4. Conclusions

In conclusion, except for the rare case of sitosterolemia, modest increases in phytosterol levels, which indicate increased cholesterol absorption, may increase an individual’s CVD risk, although inconsistency still exists among studies. Whether ezetimibe is more effective in patients with raised levels of plant sterols also needs to be proven. Therefore, there is a need for trials elucidating these issues.
